# Protein Tyrosine Phosphatase N2 Is a Positive Regulator of Lipopolysaccharide Signaling in Raw264.7 Cell through Derepression of Src Tyrosine Kinase

**DOI:** 10.1371/journal.pone.0162724

**Published:** 2016-09-09

**Authors:** Huyen Trang Ha Thi, Seo-Won Choi, Young-Mi Kim, Hye-Youn Kim, Suntaek Hong

**Affiliations:** Laboratory of Cancer Cell Biology, Lee Gil Ya Cancer and Diabetes Institute, Gachon University, Incheon, Korea; Universita degli Studi di Padova, ITALY

## Abstract

T cell protein tyrosine phosphatase N2 (PTPN2) is a phosphotyrosine-specific nonreceptor phosphatase and is ubiquitously expressed in tissues. Although PTPN2 functions as an important regulator in different signaling pathways, it is still unclear what is specific target protein of PTPN2 and how is regulated in lipopolysaccharide (LPS)-induced inflammatory signaling pathway. Here, we found that PTPN2 deficiency downregulated the expression of LPS-mediated pro-inflammtory cytokine genes. Conversely, overexpression of PTPN2 in Raw264.7 cells enhanced the expression and secretion of those cytokines. The activation of MAPK and NF-κB signaling pathways by LPS was reduced in PTPN2-knockdowned cells and ectopic expression of PTPN2 reversed these effects. Furthermore, we found that PTNP2 directly interacted with Src and removed the inhibitory Tyr527 phosphorylation of Src to enhance the activatory phosphorylation of Tyr416 residue. These results suggested that PTPN2 is a positive regulator of LPS-induced inflammatory response by enhancing the activity of Src through targeting the inhibitory phosphor-tyrosine527 of Src.

## Introduction

The immune system works specifically to protect the host against foreign threats, such as bacteria and viruses, and to remove endogenous damaged cells that are mainly controlled by the immune cells such as macrophages, neutrophils and mast cells [1]. In normal conditions, the production and activation of chemokines and cytokines such as tumor necrosis factor- (TNF-α), interleukin-6 (IL-6), IL-12 or inflammatory mediators as well as the elimination of foreign threats are tightly controlled for homeostasis maintenance [2]. However, these prolonged immune responses cause chronic inflammatory process which results in various immune-associated diseases, cancer and diabetes [3]. Recent evidences suggest that endotoxin of gram-negative bacteria, lipopolysaccharide (LPS), is one of the regulators of inflammatory response in many different cells [4]. In monocytes and macrophages, LPS activates the toll-like receptors (TLRs) resulting in the secretion of pro-inflammatory cytokines including TNF-α [5]. In the lung tissue, LPS regulates the activation of NF-κB signaling pathways that enhances the expression of pro-inflammatory genes such as TNF-α, COX-2 and ICAM-1 [6]. In addition to NF-κB signaling, LPS-induced mitogen activated protein kinase (MAPK) pathway stimulation significantly increases the production of pro-inflammatory cytokines in cardiomyocytes [7].

T cell protein phosphatase TCPTP (encoded by *Ptpn2*) is a non-receptor protein tyrosine phosphatase that is ubiquitously expressed in all stages of development [8]. PTPN2 consists of two splice variants in human and share a PTP catalytic domain at the N termini, but are different at their non-catalytic C termini. The 45 kDa form is localized in both nucleus and cytoplasm and the 48 kDa form is present in the endoplasmic reticulum. Both forms are identified in human but only the 45 kDa form is detectable in mice [9]. PTPN2 may negatively or positively regulates the intracellular signaling processes through the dephosphorylation of specific substrates. Many phosphorylated proteins are identified as the targets of PTPN2 including the signal transducers and activators of transcription 1 (STAT1), STAT3 [10], Janus activated kinase 1 (JAK1) and JAK3 [11]. Recent studies also reported that PTPN2 plays specific roles in regulation of the inflammatory signaling pathway. A strong evidence for the role of PTPN2 in the inflammatory process is that PTPN2 knockout mice develop severe inflammation with splenomegaly, lymphadenopathy and excessive production of TNF-α, IFN-γ, IL-12 and nitric oxide, then die 5 weeks after birth [12]. PTPN2 has also been identified as a key inhibitory regulator of T cell receptor (TCR) signaling pathway, which suppresses the autoimmune and inflammatory diseases through the dephosphorylation and inactivation of Src family kinases [13].

Src is a tyrosine protein kinase that controls many biological processes including cellular metabolism, survival, differentiation, migration and proliferation. Src catalytic activity is modulated by the phosphorylation and dephosphorylation of tyrosine residues. The sixth residue from the C-terminal region contains the important phosphorylation site of Src, Tyr527. This residue is a negative regulatory one which is phosphorylated 90–95% under the basal conditions and the dephosphorylation of this site promotes the Src kinase activity [14]. Conversely, the autophosphorylation site at Tyr-416 on catalytic domain is critical for the activation of Src kinase. Autophosphorylation of Tyr-416 displays an active structure of Src and dephosphorylation of Tyr416 decreases Src kinase activity [15]. Therefore, a modulation of the tyrosine residue phosphorylation is a critical process for the enzymatic activity of Src family kinases. Previous studies suggested that Src plays multiple roles in inflammatory signaling. Src kinase is involved in the macrophage-involved innate immunity marked by phagocytosis, inflammatory cytokine/mediator production, and cellular migration [16]. Moreover, recent evidence suggests that there is an involvement of c-Src activation in response to LPS signaling pathway. Src tyrosine kinases markedly enhanced LPS-induced activation of NF-κB and integrin (α_v_β_3_) signaling during acute lung injury [17]. The inhibition of Src-family kinase impaired the phosphorylation of c-Jun, resulting in the reduction of the formation of AP-1 complexes upon LPS stimulation [18]. Although many different signaling pathways are involved in the LPS-induced inflammatory response, how PTPN2 modulates the Src kinase activity upon LPS stimulation still remains unclear.

In order to determine the specific role of PTPN2 in LPS-induced inflammatory signaling, we used lentiviral shRNA knockdown and overexpressing systems for PTPN2 in macrophage, RAW264.7 cells. By using the cytokine array, we identified the differentially expressed cytokine genes and validated them with real-time quantitative PCR (qRT-PCR) and ELISA. We then found that PTPN2 promotes the LPS-induced inflammatory signaling and directly regulates Src activity by modulating the inhibitory phosphorylation at Tyr527 residue.

## Materials and Methods

### Cell culture and reagents

The murine macrophage RAW264.7 cells were cultured in DMEM (Welgene, Daegu, Korea) supplemented with 1% streptomycin/penicillin (Invitrogen, Carlsbad, CA) and 10% heat inactivated fetal bovine serum at 37°C in a CO_2_ incubator. Antibodies against phosphor-Src (Tyr416) (#2101), phosphor-Src (Tyr527) (#2105), phosphor-p44/42 (ERK1/2) (#4377), phosphor-p38 (#4631) and phosphor-NF-κB (#3033) were from Cell Signaling (Beverly, MA). Mouse anti-Myc and rabbit anti-PTPN2 antibodies were purchased from Santa Cruz Biotechnology (Santa Cruz, CA). MAP kinase inhibitors (SB203580 and U0126) were obtained from Merck Calbiochem (San Diego, CA). Src kinase inhibitor (SU6656), NF-κB inhibitor (BMS345541) and LPS were purchased from Sigma-Aldrich (St. Louis, MO).

### Cytokine PCR array and ELISA

To identify the differentially regulated cytokines and chemokines by PTPN2, the control or PTPN2-knockdowned cells were incubated for 4 hr with LPS (1 μg/ml). In addition, total RNA was extracted with TRIzol reagent (Invitrogen, Carlsbad, CA) and analyzed with mouse RT^2^ profiler PCR array in accordance with the manufacturer’s protocol (PAMM-150Z, Qiagen, Valencia, CA). To determine the cytokine levels in cultured RAW264.7 cell, supernatants were collected at 6 hr (TNF-α) or 24 hr (IL-6 and IL-1β) after LPS stimulation. ATP (3 mM) was cotreated with LPS to induce the secretion of IL-1β into the culture media. The ELISA kits for measurement of mouse IL-6, IL-1β and TNF-α were supplied from R&D Systems (Minneapolis, MN). Assays were carried out in accordance with the manufacturer’s instruction. The absorbance at 450 nm was measured using a Microplate reader (BioTek Instrument, Winooski, VT). Experiments were performed in triplicates to obtain statistical significance.

### Validation of gene expression using quantitative real-time PCR

Total RNA was extracted with TRIzol reagent and mRNA was transcribed with random hexamers using Super Script II (Invitrogen). SYBR-green Premix Ex-Tag II (Takara, Madison, WI) was used for the quantification of cytokine transcripts with a real-time quantitative PCR on an Applied Biosystem Prism 7900HT sequence detection system. PCR results were analyzed using the comparative ddCt method with cyclophilin as control. The experiments were performed in triplicates and expressed as the mean S.D. The *p* values were determined with a two-tailed *t*-test.

### Immunoblotting and immunoprecipitation

Proteins were extracted with a lysis buffer (25 mM HEPES (pH 7.5) 150 mM NaCl, 1% Triton X-100, 10% glycerol, 5 mM EDTA and a protease inhibitor cocktail) for 30 min and clarified by centrifugation at 10,000 × *g* for 10 min at 4°C. The protein concentrations were measured by the BCA method (Pierce, Rockford, IL). For immunoblotting, equal amounts of protein lysates were separated by SDS–polyacrylamide gel electrophoresis, followed by a transfer onto the polyvinylidene difluoride membrane. Membranes were treated with a blocking solution for 1 hr, which were then incubated overnight with primary antibodies. Immunoreactive proteins were checked with the chemiluminescence method after incubation with a secondary antibody according to the manufacturer’s protocol (Pierce). For immunoprecipitation, the equal amounts of protein lysates were incubated with specific antibodies at 4°C and incubated with protein A beads (Bioprogen, Daejeon, Korea) with a 3 hr rotation. The beads were washed 3 times with a washing buffer (25 mM Tris-HCl, pH 8.0, 150 mM NaCl, 1% Triton X-100) and binding proteins were eluted by adding 2X Tris-Glycine SDS sample buffer at 100°C for 5 min. The extracts were analyzed with Western blotting as described above.

### Generation of PTPN2-D182A mutant

The PTPN2-MT (D182A) is mutated at Asp 182 residue to Ala and has the similar affinity for substrate with wild-type, but the catalytic activity was reduced. Therefore, PTPN2-MT can form a stable complex with tyrosine phosphorylated substrates and protect those substrates from endogenous phosphatase-induced dephosphorylation [19]. The EZchange^TM^ site-directed mutagenesis kit was used to generate the PTPN2 substrate trapping D182A mutant (PTPN2-MT) in accordance with the manufacturer’s protocol (Enzynomics, Daejeon, Korea). The generated mutation of PTPN2 was validated with DNA sequencing.

### Generation of stable cell lines using lentivirus

Lenti HEK293T packaging cells were cultured and transfected with a pCAG lentiviral vector (GFP, Myc-tagged PTPN2-WT or PTPN2-MT) using a Lipofectamine 2000 reagent (Invitrogen). The transfected cells were maintained in DMEM which contained 10% FBS and secreted lentiviruses were collected after 48 hr using 0.45 μm filters. The Raw264.7 cells were infected with different lentiviral supernatants 3 timers every 12 hr with Polybrene (8 μg/ml) (Sigma). The expression of PTPN2 was checked by Western blotting with an anti-Myc antibody.

The silencing of endogenous mouse PTPN2 was processed using the pLKO lentiviral short hairpin RNA (shRNA) system according to the manufacturer’s protocol (Sigma). After preparing the lentiviral supernatant, the Raw264.7 cells were infected 3 times every 12 hr. After 48 hr of infection, the shRNA efficiency was measured with qRT-PCR and Western blotting.

## Results

### PTPN2 deficiency downregulates LPS-induced pro-inflammatory signaling

**Fig 1:**
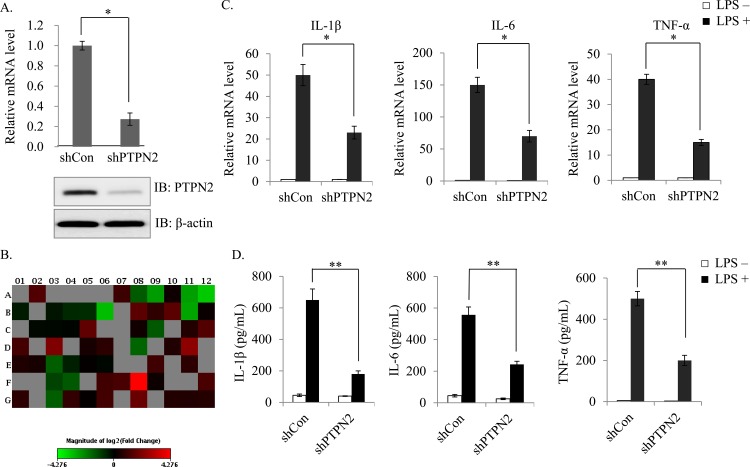
PTPN2 deficiency downregulates LPS-induced pro-inflammatory signaling. (A) PTPN2 mRNA and protein expression level in PTPN2-knockdowned cells. (B) The heat map represents the pro-inflammatory cytokine profiling of PTPN2-knockdowned cells relative to parental control Raw264.7 cells after treatment with LPS (1 μg/ml) using cytokine array. (C) The mRNA expression of IL-1β, IL-6 and TNF-α in control and PTPN2-knockdowned cells was examined by quantitative real-time PCR. (D) Secreted cytokines were measured with ELISA after 6 or 24 hr treatment of LPS. Data represent the means ± S.D. of three independent experiments. *, *p* < 0.05 and **, *p* < 0.01 (Student *t* test).

To confirm the role of PTPN2 on LPS‑induced gene expression, we made PTPN2-knockdowned stable Raw264.7 macrophage cell line using the shRNA lentiviral system. The shRNA significantly suppressed the PTNP2 mRNA and protein expression level compared with control cells (Fig 1A). Then, we performed a cytokine array to screen for LPS-stimulated pro-inflammatory cytokines which are differentially expressed between control and PTPN2-knockdowned cells. Upon LPS stimulation, several pro-inflammatory cytokines were downregulated in PTPN2-knockdowned cells including IL-1β, IL-6 and TNF-α (Fig 1B). To validate the results of cytokine array, the pro-inflammatory gene expression levels were confirmed using qRT-PCR and ELISA. We showed that IL-1β, IL-6 and TNF-α mRNA were reduced in PTPN2-knockdowned cells compared with control cells (Fig 1C) and the production of these cytokines in media supernatants was also suppressed by PTPN2 deficiency (Fig 1D). We indicated that PTPN2 positively regulates the LPS-induced IL-1β, IL-6 and TNF-α expression and secretion in the RAW264.7 cells.

PTPN2 has been known as a negative regulator of inflammatory signaling in the interferon gamma (IFN-γ)-treated THP-1 cells or inflammatory bowel disease [20]. To confirm the specificity of our study, we checked the LPS or IFN-γ-induced signaling pathways in THP-1 or J774A.1 cells. Like LPS-stimulated RAW264.7 cell, PTPN2 depletion reduced the expression of inflammatory cytokines in the mouse J774A.1 and human THP-1 monocyte cells after treatment with LPS (S1A, S1B and S2A Figs). Interestingly, previous studies suggested that the loss of PTPN2 promoted the expression of IFN-γ-induced pro-inflammatory cytokines in THP-1 cells [21]. Consistent with previous results, the knockdown of PTPN2 in Raw264.7 and THP-1 cells enhanced the expression of IFN-γ-induced pro-inflammatory genes (S1C and S1D Fig) and secretion of these cytokines (S2B Fig). We also tested the effect of PTPN2 on LPS or IFN-γ-induced MAPK and NF-κB activation as well as the regulation of Src phosphorylation in several different cell lines. As shown in S3A and S3B Fig, the loss of PTPN2 in J774A.1 and THP-1 cells suppressed the phosphorylation of Src at Tyr 416 and the activation of ERK, p38 and NF-κB signaling upon LPS stimulation, like in Raw264.7 cells (S3A and S3B Fig). However, consistent with previous reports, the depletion of PTPN2 in Raw264.7 and THP-1 cells enhanced the activation of MAPK and NF-κB signaling pathways and phosphorylation of Src at Tyr 416 residue upon treatment with IFN-γ. These results strongly suggest that the regulation of phosphotyrosine status of Src by PTPN2 is dependent on a specific stimulus.

### PTPN2 overexpression enhances LPS-stimulated pro-inflammatory signaling

**Fig 2:**
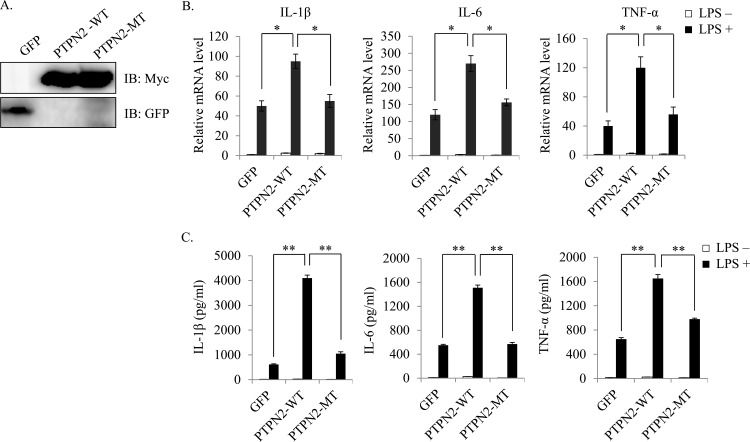
Overexpression of PTPN2 enhances LPS-stimulated cytokine genes. (A) The expression of PTPN2-WT and PTPN2-MT (substrate trapping mutant) was detected with anti-Myc antibody and control cell was confirmed by GFP expression. (B-C) GFP, PTPN2-WT and PTPN2-MT cells were treated with LPS (1 μg/ml) for 4 hr to check the mRNA expression or for 24 hr to measure the cytokine secretion. IL-1β, IL-6 and TNF-α mRNA expression levels were examined by qRT-PCR (B) and secreted cytokines were measured with ELISA (C). Data represent the means ± S.D. of triplicate experiments. *, *p* < 0.05 and **, *p* < 0.01 (Student *t* test).

To verify the effect of PTPN2 on LPS-stimulated pro-inflammatory cytokine expression, we generated the PTPN2 wild-type (PTPN2-WT) or PTPN2 mutant (PTPN2-MT) Raw264.7 cell lines using lentiviral system The PTPN2-MT (D182A) is a substrate trapping mutant that is mutated at the Asp 182 residue to Ala, which has a similar affinity for a substrate with the wild-type; but the catalytic activity was reduced. Therefore, PTPN2-MT can form a stable complex with the tyrosine phosphorylated substrates, protecting those substrates from endogenous phosphatase-induced dephosphorylation [19]. As shown in Fig 2A, PTPN2-WT and PTPN2-MT were well detected with an anti-Myc antibody. To address the specific role of PTPN2 in LPS signaling, PTPN2-WT and PTPN2-MT cells were starved for 4 hr and then stimulated with LPS (1 μg/ml) for 4 hr or 24 hr. Upon stimulation of LPS, PTPN2-WT significantly enhanced both mRNA and secreted protein levels of those cytokines which were suppressed in the absence of PTPN2 (Fig 2B and 2C). Conversely, PTPN2-MT did not enhance the pro-inflammatory cytokine expression induced by LPS (Fig 2B and 2C). Together, these data suggest that PTPN2 positively regulates LPS-induced inflammatory signaling through its intact phosphatase activity in Raw264.7 cell.

### PTPN2 promotes LPS-induced cytokine expression though MAPK and NF-κB regulation

**Fig 3:**
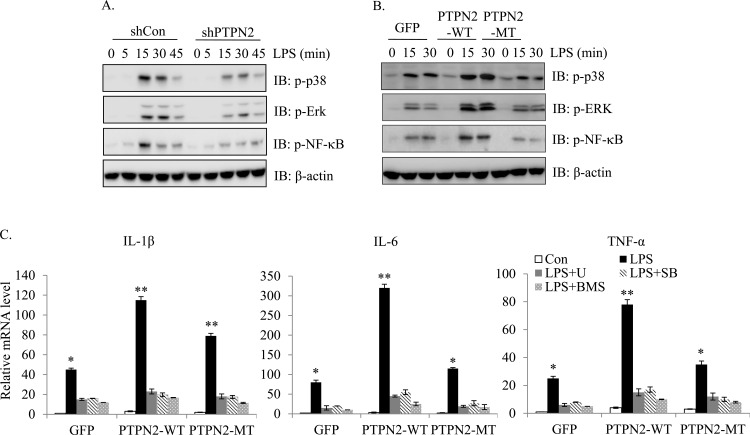
PTPN2 promotes LPS signaling through activation of MAPK and NF-κB. (A-B) Scramble and PTPN2-knockdowned cells (A) or control, PTPN2-WT and MT cells (B) were stimulated with LPS (1 μg/ml) for various durations. Immunoblotting was performed with specific antibodies to detect the phosphorylation of MAPK and NF-κB. The β-actin was used as the internal control. (C) MAPK inhibitors (U or SB, 10 μM) and NF-κB inhibitor (BMS, 10 μM) were pre-incubated for 2 hr, followed by the treatment of LPS (1 μg/ml) for 4 hr. IL-1β, IL-6 and TNF-α mRNA expression levels were determined by qRT-PCR. Data represent the means ± S.D. of three independent experiments. *, p < 0.05 and **, p < 0.01 (Student *t* test).

A previous report has shown that LPS enhances the pro-inflammatory response through the phosphorylation of extracellular receptor kinases (ERK), p38 and nuclear translocation of NF-κB [22]. Therefore, we hypothesized that PTPN2 may regulates the LPS-induced cytokine productions through enhancing the activity of MAPK and NF-κB signaling pathway. We first determined the effect of PTPN2 on the activation of MAPK and NF-κB signaling pathway. As expected, the treatment of LPS (1 μg/ml) rapidly increased the phosphorylation of p38, ERK, and NF-κB after 15 min, which was then gradually decreased after 30 min. However, the PTPN2-knockdowned cells significantly blocked the phosphorylation of p38, ERK and NF-κB in comparison with control cells (Fig 3A). Conversely, the ectopic expression of PTPN2-WT markedly enhanced the activation of p38, ERK and NF-κB (Fig 3B). However, the loss of phosphatase activity blocked the positive activity of PTPN2 in LPS signaling.

We next confirmed the involvement of p38, ERK and NF-κB activity in the production of inflammatory cytokines by PTPN2. PTPN2 overexpressing RAW264.7 cells or control cells were pre-incubated with vehicle DMSO or ERK inhibitor (U0126), p38 inhibitor (SB203580), and NF-κB inhibitor (BMS345541) for 2 hr, followed by a treatment of LPS (1 μg/ml) for 4 hr. As shown in Fig 3C, the expression of inflammatory genes was significantly suppressed by pre-treatment of ERK, p38 or NF-κB inhibitors. These results confirmed that PTPN2 positively regulates LPS-induced cytokine expression dependent on p38, ERK and NF-κB signaling pathways, and shows that it functions at the upstream of these pathways.

### PTPN2 associates and activates Src kinase through dephosphorylation of Tyrosine 527

**Fig 4:**
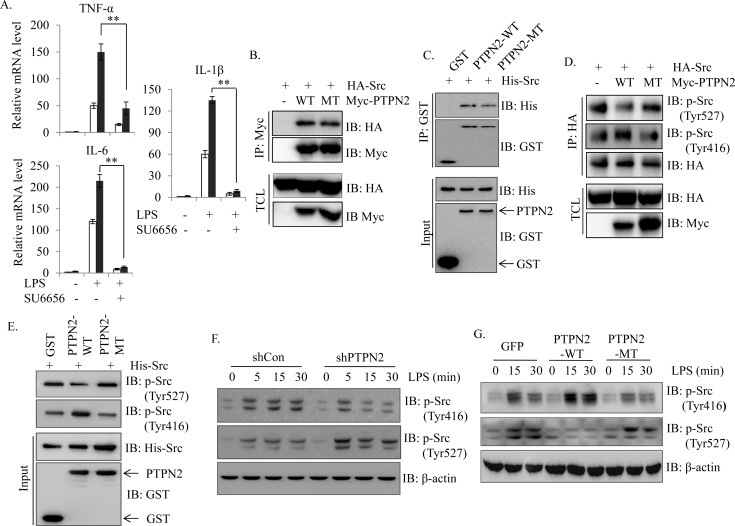
PTPN2 associates with Src and modulates phosphorylation pattern of Src. (A) Control (□) or PTPN2-WT (■) cells were pre-incubated with Src inhibitor (SU6656) (10 μM) or vehicle for 2 hr and then stimulated with LPS for 4 hr. IL-1β, IL-6 and TNF-α mRNA expression levels were examined by qRT-PCR and normalized with cyclophilin. Data represent the means ± S.D. of three independent experiments. **, *p*< 0.01 (Student *t* test). (B) 293T cells were transiently cotransfected with HA-Src and control, Myc-PTPN2-WT or Myc-PTPN2-MT. After 36 hr post transfection, equal amount of protein lysates was immunoprecipitated with an anti-Myc antibody, followed by immunoblotting with anti-HA (Src) and anti-Myc antibodies. Total cell lysates were immunoblotted with anti-HA and anti-Myc antibodies. (C) Recombinant GST or GST-PTPN2 was incubated with equal amounts of His–Src fusion protein. After pulldown with GSH-beads, the bound proteins were immunoblotted with an anti-His antibody. GST and GST-PTPN2 were detected by immunoblotting with an anti-GST antibody. (D) HA-Src and control, Myc-PTPN2-WT or Myc-PTPN2-MT were transiently cotransfected into the 293T cells. After 36 hr post transfection, protein lysates were immunoprecipitated with anti-HA antibody, followed by immunoblotting with specific antibodies against phosphor-Src at different residues. (E) To confirm the direct regulation of Src by PTPN2, an *in vitro* phosphorylation assay was performed. Recombinant Src and PTPN2 were incubated for 1 hr in the presence of ATP, and the phosphorylated status of Src was checked with phosphor-specific antibodies. (F-G) PTPN2-knockdowned cells (F) or overexpressed cells (G) were stimulated with LPS (1 μg/ml) for several times. Protein lysates were analyzed with related specific antibodies to confirm the phosphorylation pattern of Src.

To identify the target protein of PTPN2, we selected Src kinase as a potential candidate that can serve as a physiological substrate for PTPN2 in response to LPS stimulation. First, we examined the involvement of Src in LPS-induced inflammatory response by the treatment of a Src specific inhibitor (SU6656). As shown in Fig 4A, the inhibition of Src with SU6656 not only suppressed the LPS-induced pro-inflammatory gene expression in both PTPN2-WT and control cells, but also significantly reduced the promotive activity of PTPN2 in LPS-induced inflammatory response. These results suggested that PTPN2 acts as an upstream regulator of Src in response to LPS stimulation. To confirm that Src is a target of PTPN2, we examined the interaction between Src and PTPN2. As shown in Fig 4B, both PTPN2-WT and PTPN2-MT clearly interacted with Src protein. To further confirm the direct interaction, we performed an *in vitro* binding assay with recombinant PTPN2 and Src proteins. We found that both PTPN2-WT and PTPN2-MT directly interacted with Src protein (Fig 4C). We also performed the immunofluorescence staining to further confirm the interaction of PTPN2 and Src within the substructure of cells. As shown in S4 Fig, both PTPN2-WT and PTPN2-MT were colocalized with Src in transfected cells. Over 80% of PTPN2 were overlapped with Src in cotransfected cells. These results strongly support that PTPN2 directly interacted and colocalized with Src.

We thought that PTPN2 may regulate Src through the dephosphorylation of phosphor-Tyr at 527 residue. To prove this hypothesis, Src was immunoprecipitated and then immnunoblotted with anti-phospho-Src antibodies (Tyr416 or Tyr527). Interestingly, we observed a decrease of phosphorylated Src at Tyr527 residue in the presence of PTPN2-WT, but not PTPN2-MT. Conversely, PTPN2-WT enhanced the phosphorylation of Src at Tyr416 residue compared with control and PTPN2 mutant (Fig 4D). We also performed an *in vitro* phosphorylation assay to examine whether PTPN2 directly activates Src protein. Purified His-Src was incubated with the beads containing the GST-PTPN2 fusion protein for 60 min in the presence of ATP. Then, the Src proteins were immunoblotted with anti-phospho-Src (Tyr527) or phospho-Src (Tyr416) antibodies. PTPN2-WT-but not PTPN2-MT-significantly increased the phosphorylation of Src at Tyr416 residue and decreased the phosphorylated Src at Tyr527 residue (Fig 4E). We showed that PTPN2 could directly target the Src protein to regulate the downstream signaling through the enhancement and reduction of the phosphorylation of Src at Tyr416 and Tyr527 residues, respectively.

To further confirm the differential phosphorylation of Src by PTPN2 in LPS signaling, we explored whether the expression of PTPN2 regulates the Tyr phosphorylation of Src at the 416 and 527 sites. As expected, we found that the phosphorylation of Src (Tyr 416) was markedly inhibited in PTPN2-knockdowned RAW264.7 cells compared with control cells, but the phosphorylation of Src (Tyr 527) was significantly enhanced in stimulation of LPS (Fig 4F). In contrast, the expression of PTPN2-WT-but not PTPN2-MT-enhanced the phosphorylation of Src (Tyr 416) and dephosphorylation of Src (Tyr 527) compared with control cells after the treatment with LPS (Fig 4G). Taken together, we have shown that PTPN2 modulates LPS-induced inflammatory response through the differential regulation of Src Tyrosine phosphorylation at different residues-either Tyr416 or Tyr527-in Raw264.7 cell.

## Discussion

**Fig 5:**
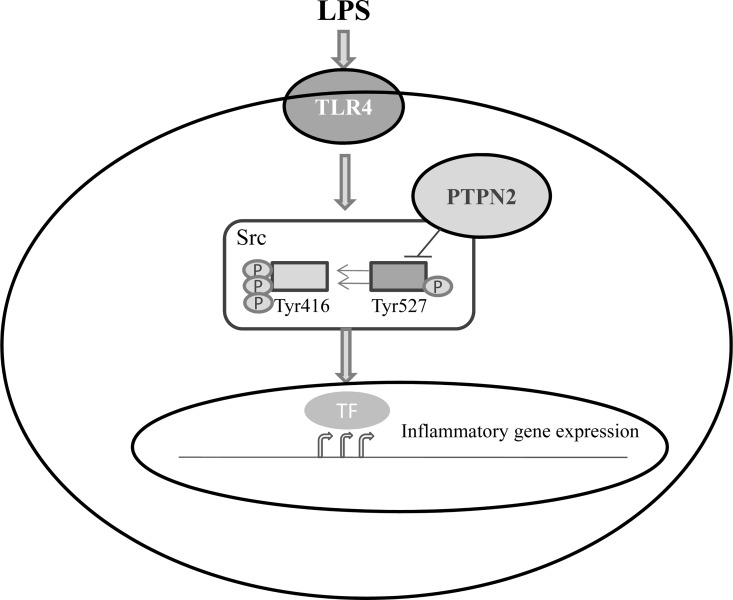
Schematic diagram showing the involvement of PTPN2 in LPS-induced inflammatory signaling through derepression of Src. Upon LPS stimulation, PTPN2 directly interacts with Src, and promoting the activation of Src kinase through dephosphorylation of Tyr527 residue, and in turn enhances phosphorylation of Tyr416. The activation of Src enhances the LPS-mediated expression of pro-inflammatory cytokine genes through MAPK and NF-κB signaling.

PTPN2 is well known to be an important negative regulator of various cellular signaling pathways through the dephosphorylation of adaptor proteins [23][24]. In contrast, we found a positive action of PTPN2 in the inflammatory signaling pathway through the activation of c-Src in this study. Herein, we showed that PTPN2 directly interacts with Src, targeting phosphor-Tyr527 for dephosphorylation, which results in an increase of Src downstream signaling in Raw264.7 cell (Fig 5). Previously, CD45 (another tyrosine phosphatase) has been demonstrated to control the activation of Src family kinases by the dephosphorylation of the C-terminal CSK inhibitory phosphorylation site of lymphocyte [25]. Conversely, PTPN2 inhibited the activation of Src signaling though the dephosphorylation of the active phosphorylated site on Src family kinases in hematopoietic cells, but not in splenocytes [26]. On the other hand, PTPN2 that was associated with TRAF2 dephosphorylated and inhibited Src to selectively regulate ERK signaling in response to TNF-α, but not in response to other stimuli such as EGF and PDGF [10]. Moreover, the overexpression of PTPN2 suppressed the anti-CD3ε-induced SFK Y418 phosphorylation and downstream ERK signaling in T cells through the dephosphorylation of SFK Y418 [27]. In this study, we identified that PTPN2 differentially regulates the phosphorylation of Src and downstream signaling depending on the stimuli (Figs 1 and 2, S1 and S2 Figs). These results may originate from the interaction of PTPN2 with a different domain of Src or the recruitment of specific adapter proteins on the PTPN2-Src complex to show differential regulation of Src phosphorylation. These results strongly suggest that the regulation of phosphotyrosine status of Src by PTPN2 and other protein tyrosine phosphatases is a stimuli-specific phenomenon. Further studies are required to identify a more detailed mechanism.

Our results also indicated that PTPN2 regulates the LPS-induced inflammatory gene expression through the activation of ERK, p38, and NF-κB signaling pathways (Fig 3). The treatment of ERK, p38 and NF-κB inhibitors suppressed the LPS-induced inflammatory gene expression, which demonstrates an involvement of ERK, p38 and NF-κB signaling pathways in PTPN2-mediated LPS signaling (Fig 3C). Because NF-κB is phosphorylated at the Serine residue and PTPN2 is a Tyrosine phosphatase, the effect of PTPN2 on the phosphorylation of NF-κB may not be a direct interaction. However, Src kinase is a well-known upstream regulator for both NF-κB and MARK signaling pathway [17]. Therefore, we hypothesized that PTPN2 could have a direct effect on the phosphorylation of tyrosine residues of Src kinase, indirectly modulating the activation of Src downstream signaling including the NF-κB, ERK and p38 signaling pathways. Although the functional correlation between PTPN2, Src and NF-κB signaling pathways has not been fully characterized thus far, the involvement of Src in LPS-induced inflammatory signaling was confirmed by the treatment of Src inhibitor (Fig 4A). These results supported that the presence of PTPN2 enhanced ERK, p38 and NF-κB through the activation of Src in response to LPS in RAW264.7 cells. Moreover, further investigations are necessary to determine whether the positive role of PTPN2 is applicable for *in vivo* macrophages or dependent on the cell-type or species. In conclusion, we propose that PTPN2 provides a platform for Src activation resulting in the enhancement of LPS signaling pathway. In addition, it will be very interesting to examine the effect of PTPN2 in the inflammation and inflammation-associated diseases via controlling PTPN2 in macrophages.

## Supporting Information

S1 Doc Immunofluorescence microscopy. HEK 293 cells were seeded on 8-well plate and cotransfected either with myc-PTPN2-WT or myc-PTPN2-MT and HA-Src. After 48 hr transfection, cells were fixed with 4% (v/v) paraformaldehyde for 20 min at room temperature, and then permeabilized with 0.5% Triton X-100 in phosphate-buffered saline. Next, the cells were stained with Myc or HA primary antibodies and subsequently with Alexa Fluor 488-conjugated goat anti-mouse secondary antibody or Alexa Fluor 594-conjugated goat anti-rabbit secondary antibody (Invitrogen). Cell nuclei were stained with 40,6-diamidino-2-phenylindole (DAPI). After staining, fluorescence images were acquired using a LSM700 confocal microscope (Carl Zeiss, Thornwood, NY). Colocalization of Src and PTPN2 was quantified with ZEN analysis software in cotransfected cells.
(DOCX)

S1 Fig PTPN2 deficiency differentially regulates LPS and IFN-γ-induced cytokine expression in monocytes. The mRNA expression of IL-1β, IL-6 and TNF-α in control and PTPN2-knockdowned cells was examined by quantitative real-time PCR. (A, B) PTPN2-knockdowned THP-1 (A) and PTPN2-knockdowned J774A.1 (B) cells suppressed the LPS-induced IL-1β, IL-6 and TNF-α expression. (C, D) PTPN2-knockdowned Raw264.7 (C) and PTPN2-knockdowned THP-1 (D) enhanced the IFN-γ-induced pro-inflammatory signaling. Data represent the means ± S.D. of three independent experiments. *, *p* < 0.05 and **, *p* < 0.01 (Student *t* test).
(TIF)

S2 Fig PTPN2 deficiency regulates LPS and IFN-γ-induced cytokine secretion in monocytes. Secreted cytokines were measured with ELISA after 24 hr treatment of LPS or IFN-γ. The graphs show secretion of IL-1β, IL-6 and TNF-α in Raw264.7 treated with IFN-γ (100 ng/ml) (A) THP-1 treated with LPS (1 μg/ml) (B). Data represent the means ± S.D. of three independent experiments. *, *p* < 0.05 and **, *p* < 0.01 (Student *t* test).
(TIF)

S3 Fig PTPN2 differentially regulates the activation of adaptor proteins upon LPS or IFN-γ stimulation. Scramble and PTPN2-knockdowned cells were stimulated with LPS (1 μg/ml) (A, B) or IFN-γ (100 ng/ml) (C, D) for indicated times. Immunoblotting was performed with specific antibodies to detect the activation of MAPK, Src and NF-κB proteins. The β-actin was used as internal control.
(TIF)

S4 Fig PTPN2 and Src are colocalized in cytoplasm. (A) HEK293 cells were co-transfected either with Myc-PTPN2-WT or Myc-PTPN2-MT (green) and HA-Src (red) for 48 hr prior to visualization by confocal microscopy. Nuclei were stained with DAPI. Colocalization of PTPN2 and Src was visualized in yellow. (B) Colocalization of Src and PTPN2-WT or PTPN2-MT was quantified with ZEN analysis software. Data represent the means ± S.D. of five optical fields. Scale bars indicate 20 μm.
(TIF)
