# Supplementary material for: Protein Tyrosine Phosphatase N2 Is a Positive Regulator of Lipopolysaccharide Signaling in Raw264.7 Cell through Derepression of Src Tyrosine Kinase
Source: PLoS One. 2016 Sep 9;11(9):e0162724. doi: 10.1371/journal.pone.0162724 (PMC5017706; doi:10.1371/journal.pone.0162724)
Supplement: S1 Doc — HEK 293 cells were seeded on 8-well plate and cotransfected either with myc-PTPN2-WT or myc-PTPN2-MT and HA-Src. After 48 hr transfection, cells were fixed with 4% (v/v) paraformaldehyde for 20 min at room temperature, and then permeabilized with 0.5% Triton X-100 in phosphate-buffered saline. Next, the cells were stained with Myc or HA primary antibodies and subsequently with Alexa Fluor 488-conjugated goat anti-mouse secondary antibody or Alexa Fluor 594-conjugated goat anti-rabbit secondary antibody (Invitrogen). Cell nuclei were stained with 40,6-diamidino-2-phenylindole (DAPI). After staining, fluorescence images were acquired using a LSM700 confocal microscope (Carl Zeiss, Thornwood, NY). Colocalization of Src and PTPN2 was quantified with ZEN analysis software in cotransfected cells. (DOCX) [file pone.0162724.s001.docx]

**Immunoﬂuorescence microscopy**

HEK 293 cells were seeded on 8-well plate and cotransfected either with myc-PTPN2-WT or myc-PTPN2-MT and HA-Src. After 48 hr transfection, cells were ﬁxed with 4% (v/v) paraformaldehyde for 20 min at room temperature, and then permeabilized with 0.5 % Triton X-100 in phosphate-buffered saline. Next, the cells were stained with Myc or HA primary antibodies and subsequently with Alexa Fluor 488-conjugated goat anti-mouse secondary antibody or Alexa Fluor 594-conjugated goat anti-rabbit secondary antibody (Invitrogen). Cell nuclei were stained with 40,6-diamidino-2-phenylindole (DAPI). After staining, ﬂuorescence images were acquired using a LSM700 confocal microscope (Carl Zeiss, Thornwood, NY). Colocalization of Src and PTPN2 was quantified with ZEN analysis software in cotransfected cells.
