# Supplementary figures and images for: Protein Tyrosine Phosphatase N2 Is a Positive Regulator of Lipopolysaccharide Signaling in Raw264.7 Cell through Derepression of Src Tyrosine Kinase
Source: PLoS One. 2016 Sep 9;11(9):e0162724. doi: 10.1371/journal.pone.0162724 (PMC5017706; doi:10.1371/journal.pone.0162724)

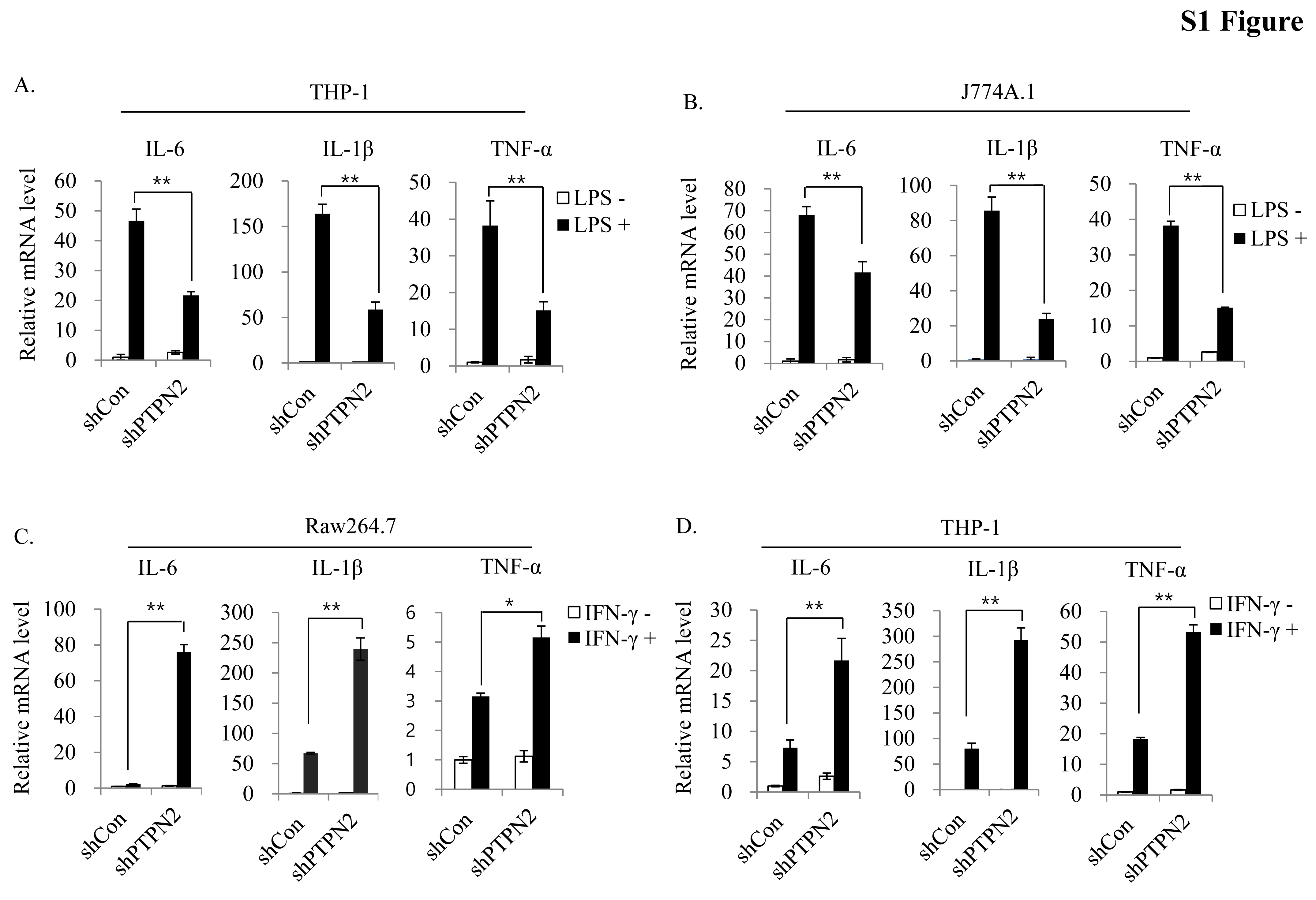

Supplement: S1 Fig — The mRNA expression of IL-1β, IL-6 and TNF-α in control and PTPN2-knockdowned cells was examined by quantitative real-time PCR. (A, B) PTPN2-knockdowned THP-1 (A) and PTPN2-knockdowned J774A.1 (B) cells suppressed the LPS-induced IL-1β, IL-6 and TNF-α expression. (C, D) PTPN2-knockdowned Raw264.7 (C) and PTPN2-knockdowned THP-1 (D) enhanced the IFN-γ-induced pro-inflammatory signaling. Data represent the means ± S.D. of three independent experiments. *, p < 0.05 and **, p < 0.01 (Student t test). (TIF) [file pone.0162724.s002.tif]

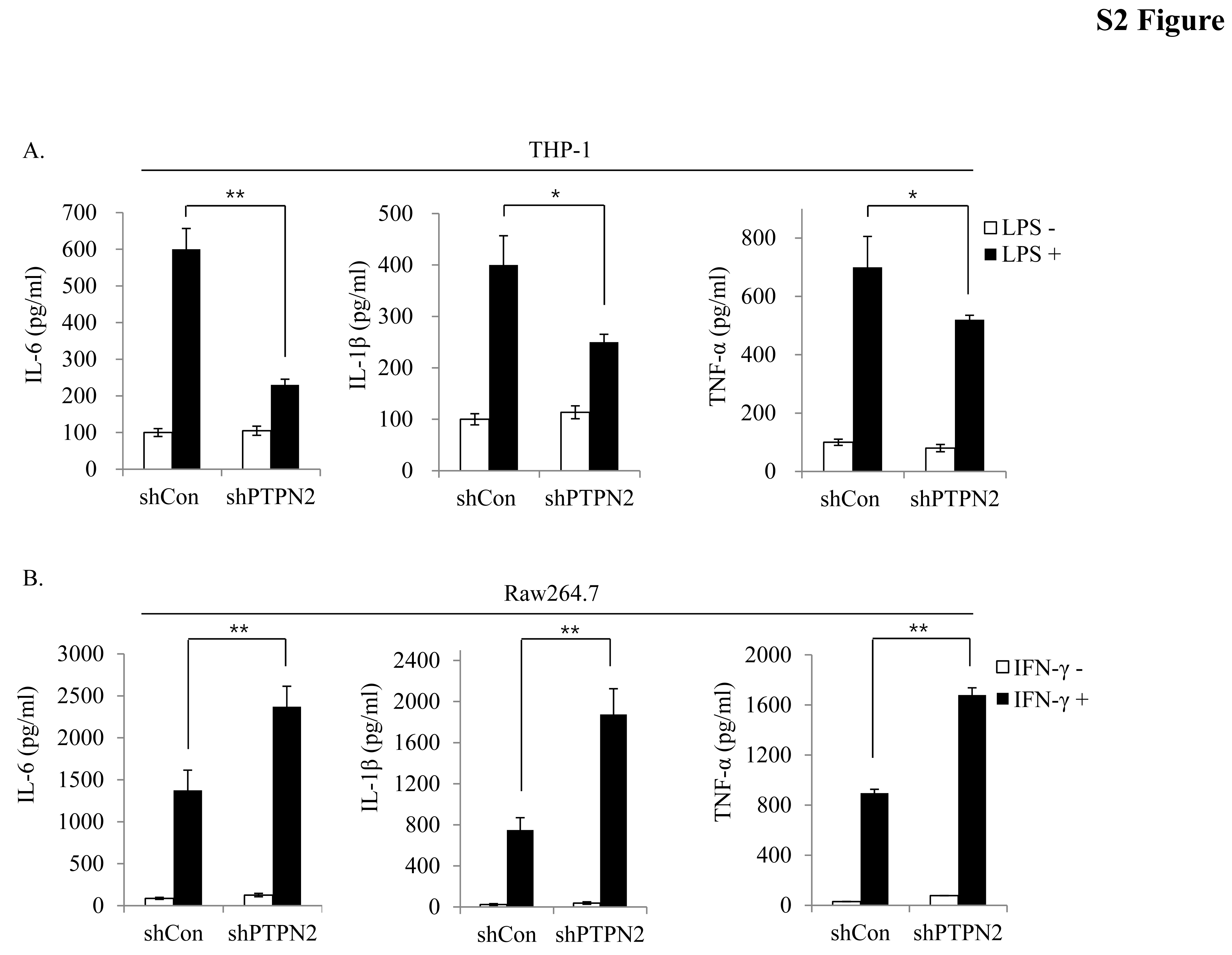

Supplement: S2 Fig — Secreted cytokines were measured with ELISA after 24 hr treatment of LPS or IFN-γ. The graphs show secretion of IL-1β, IL-6 and TNF-α in Raw264.7 treated with IFN-γ (100 ng/ml) (A) THP-1 treated with LPS (1 μg/ml) (B). Data represent the means ± S.D. of three independent experiments. *, p < 0.05 and **, p < 0.01 (Student t test). (TIF) [file pone.0162724.s003.tif]

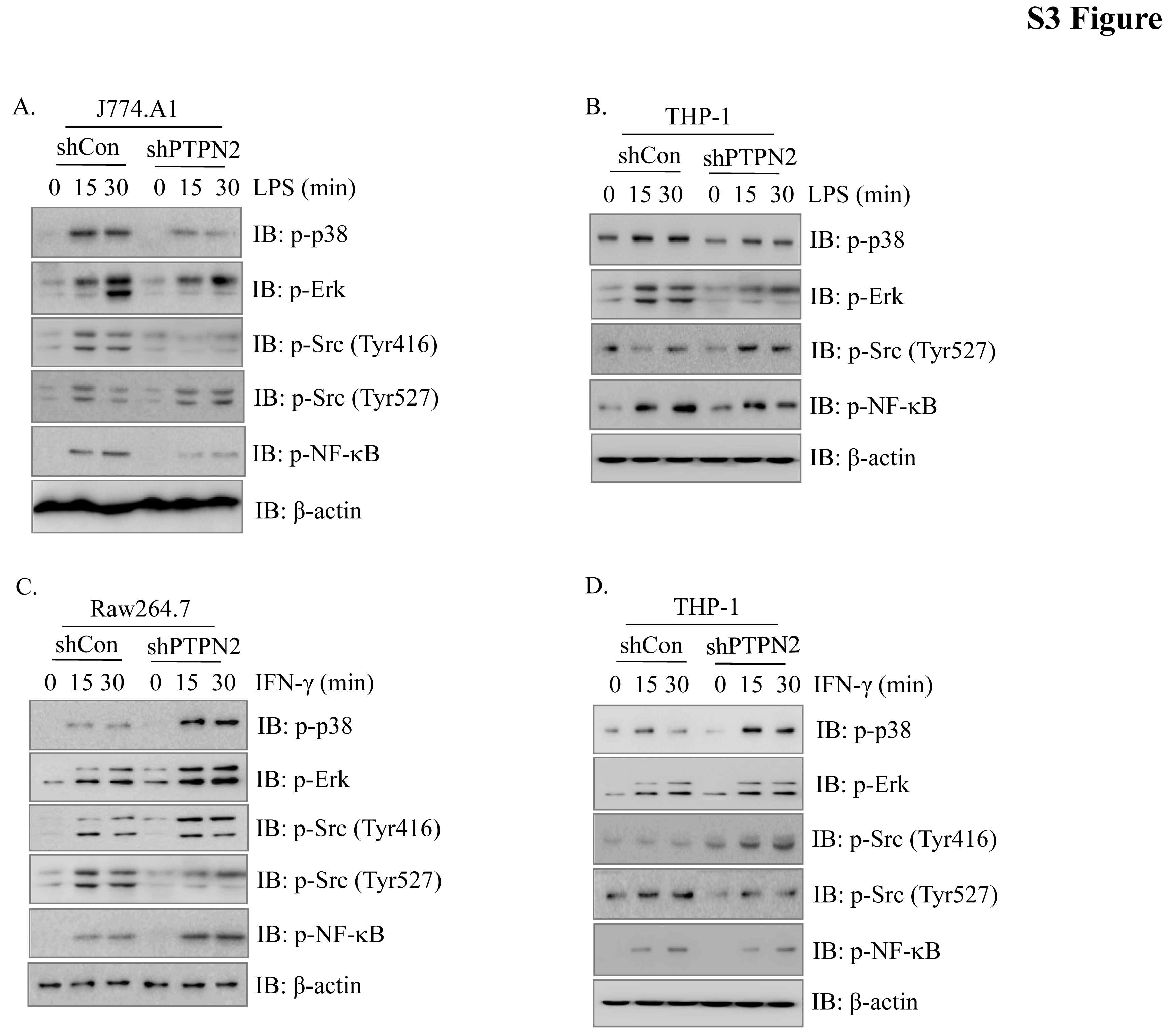

Supplement: S3 Fig — Scramble and PTPN2-knockdowned cells were stimulated with LPS (1 μg/ml) (A, B) or IFN-γ (100 ng/ml) (C, D) for indicated times. Immunoblotting was performed with specific antibodies to detect the activation of MAPK, Src and NF-κB proteins. The β-actin was used as internal control. (TIF) [file pone.0162724.s004.tif]

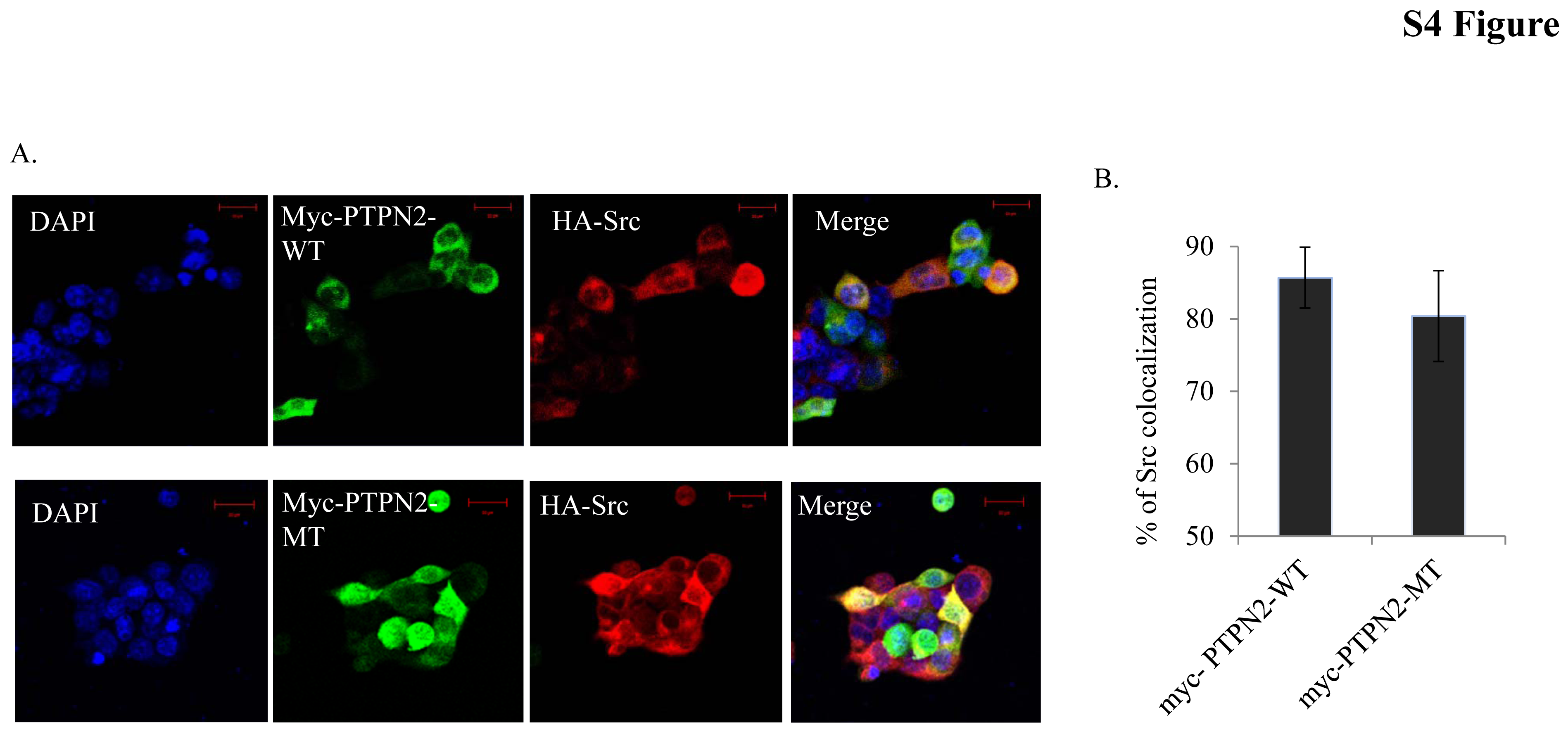

Supplement: S4 Fig — (A) HEK293 cells were co-transfected either with Myc-PTPN2-WT or Myc-PTPN2-MT (green) and HA-Src (red) for 48 hr prior to visualization by confocal microscopy. Nuclei were stained with DAPI. Colocalization of PTPN2 and Src was visualized in yellow. (B) Colocalization of Src and PTPN2-WT or PTPN2-MT was quantified with ZEN analysis software. Data represent the means ± S.D. of five optical fields. Scale bars indicate 20 μm. (TIF) [file pone.0162724.s005.tif]
